# Dry Eye Disease: Oxidative Stress on Ocular Surface and Cutting‐Edge Antioxidants

**DOI:** 10.1002/gch2.202500068

**Published:** 2025-05-14

**Authors:** Rong Hu, Jian Shi, Can‐Ming Xie, Xiao‐Lei Yao

**Affiliations:** ^1^ Department of Ophthalmology The First Affiliated Hospital of Hunan University of Chinese Medicine Changsha 410007 China; ^2^ Hunan University of Chinese Medicine Changsha 410007 China

**Keywords:** antioxidant, dry eye, nanoenzyme, oxidative damage, tear film

## Abstract

Dry eye disease (DED), a multifactorial ocular surface disorder characterized by tear film instability, is pathologically linked to oxidative damage. The accumulation of reactive oxygen species (ROS) across ocular tissues not only directly damages nucleic acids, proteins, and lipids, but also functions as an upstream driver of inflammation and tear hyperosmolarity, collectively disrupting cellular homeostasis. This review comprehensively delineates the mechanistic interplay between oxidative stress (OS) and DED pathogenesis, synthesizing evidence on enzymatic/ nonenzymatic antioxidant alterations in samples of corneal, lacrimal, conjunctival, meibomian gland, and tear tissues, alongside quantitative profiling of OS biomarkers, such as 4‐hydroxynonenal (4HNE), malondialdehyde (MDA), 8‐hydroxy‐2′‐deoxyguanosine (8‐OHdG), and 3‐nitrotyrosine (3‐NT). Furthermore, the therapeutic mechanisms of clinically approved and investigational antioxidants, including SKQ1, rebamipide, mitoquinone, elamipretide, lactoferrin, nanozymes, graphene quantum dots, tetrahedral frame of nucleic acids, and Chinese medicine monomers are critically evaluated. With the changes in the modern social environment and lifestyle, the influence of OS on DED is gradually expanding. Antioxidant‐based interventions are poised to become cornerstone components of multimodal DED management strategies.

## Graphical abstract

This review has systematically evaluated the oxidative damage of cornea, tear film, lacrimal gland, conjunctiva and meibomian gland for dry eye, and concluded the antioxidants that are either commercially available or in the research phase. GQDs: Graphene quantum dots; LF: lactoferrin; ROS: Reactive oxygen species; SOD: Superoxide dismutase; tFNA: Tetrahedral frame of nucleic acids.

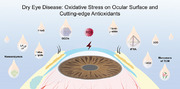

## 1 Background

**Figure 1:**
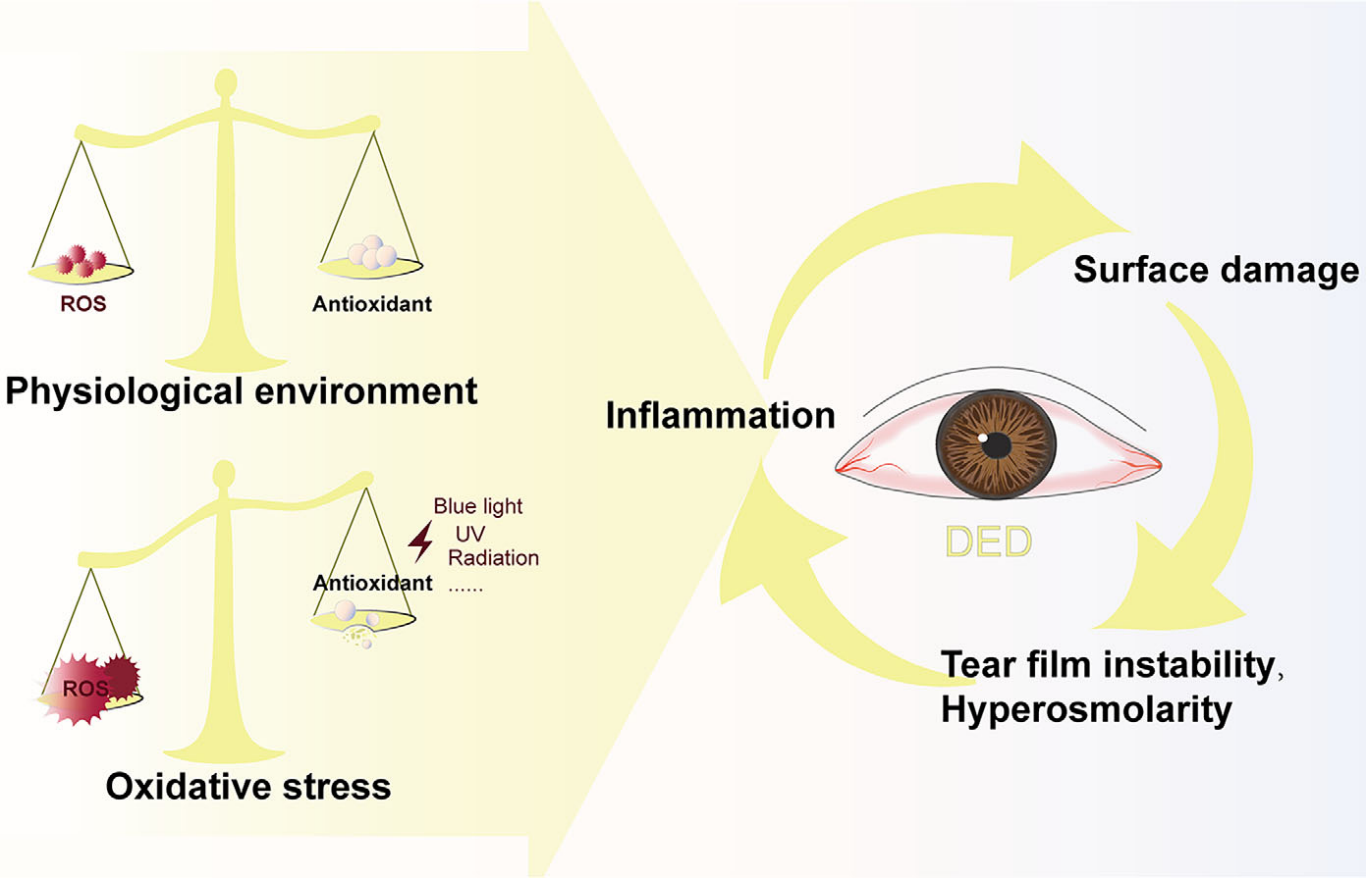
Oxidative Stress induces DED.

Dry eye disease (DED) is a multifactorial disorder typically presenting with ocular surface inflammation, epithelial lesions, and neurosensory abnormalities. The manifestations include chronic ocular dryness, visual fatigue, foreign body sensation, and neuropathic pain.^[^ ^1^ ^]^ Global epidemiological data from the International Dry Eye Workshop II indicate a 30% prevalence of DED in populations aged ≥50 years. Moreover, with the widespread application of visual display terminals, increased environmental pollution, and ultraviolet radiation, the incidence of DED has witnessed a remarkable surge, which exceeds one billion.^[^ ^2^, ^3^ ^]^ Current therapeutic paradigms prioritize symptom alleviation through ocular surface lubrication and pathomechanistic targeting via anti‐inflammatory agents. However, although Cyclosporine A (CsA) has been approved by the Food and Drug Administration (FDA) for DED treatment, 25% of patients still develop complications, including corneal epithelial damage and irritation on the periorbital skin.^[^ ^4^, ^5^ ^]^ The long‐term use of corticosteroid eye drops such as EYSUVIS also poses risks of glaucoma and cataracts.^[^ ^6^ ^]^ Drugs solely reducing inflammation seem to have limited efficacy in treating DED.^[^ ^4^, ^7^ ^]^ If inflammation is a key element that interacts with tear hyperosmolarity (THO) and surface damage, then oxidative stress (OS) could be a trigger (**Figure** **1**).^[^ ^7^ ^]^ Accumulation of reactive oxygen species (ROS) can not only directly damage nucleic acids, proteins, and lipids, disrupting cell metabolism and function, but also serve as an upstream inducer for inflammation, driving the DED process.^[^ ^4^, ^8^, ^9^ ^]^ Unfortunately, the inflammatory milieu also favors the generation of ROS.^[^ ^10^ ^]^ Therefore, emphasizing ROS scavenging and developing antioxidant and combined anti‐inflammatory and antioxidant regimens is crucial for breaking the vicious cycle.

Among the currently published review articles, there are none that analyze the relationship between OS and DED from the perspectives of various structures in the ocular surface (including the cornea, tear film (TF), lacrimal gland (LG), conjunctiva, and meibomian gland (MG）). Damage of different tissues under ROS and the changes in related biomarkers remain unclear. Therefore, in this review, the details will be mentioned. Additionally, we also summarized the current antioxidants for DED that are either commercially available or in the research phase, including SKQ1, rebamipide, mitoquinone, elamipretide, lactoferrin, nanozymes, traditional Chinese medicine monomers, and others. We anticipate that it can provide a reference for DED research, and offer novel perspectives and directions for both the development of future drugs and the management of DED.

## 2 Oxidative Stress and Alterations in DED Samples

In biology, OS denotes a pathological process arising from the imbalance between the oxidative and antioxidant systems. Humans generate energy by using oxygen as fuel, and a series of ROS will be produced during cell metabolism, including superoxide anion (O_2_^−^), hydroxyl radical (OH^•^), and hydrogen peroxide (H_2_O_2_). These by‐products can induce redox reactions and thus possess the potential to attack biological macromolecules.^[^ ^11^, ^12^ ^]^ Owing to the natural antioxidant defense mechanisms of humans, they are maintained at low levels and have a short lifespan. The ROS within the physiological range can function as part of the immune system to defend against foreign substances and safeguard regulatory proteins involved in cell signaling.^[^ ^13^, ^14^ ^]^ However, excessive ROS can trigger peroxidative damage to deoxyribonucleic acid (DNA), lipids, and proteins, resulting in alterations in cell membrane permeability, loss of enzyme activity, and the production of cytotoxic compounds, thereby affecting cell function. Eventually, the antioxidant system is overwhelmed by ROS, initiating various inflammatory and apoptotic signaling pathways and giving rise to diseases.^[^ ^14^ ^]^

Although the mechanism of DED has not been fully elucidated yet, an increasing number of studies have shown that it is closely related to the OS change. The ocular surface is constantly exposed to aerial oxygen, visible light, ultraviolet radiation, and environmental persistent free radicals. Such stimuli directly generate ROS in vitro, putting the body in an OS state and inducing the production of ROS in vivo.^[^ ^15^ ^]^ Mitochondria are the main source of endogenous ROS in cells.^[^ ^16^ ^]^ Besides, the endoplasmic reticulum, NADPH oxidase (NOX), and xanthine oxidase also contribute to it. The electron leakage from the mitochondrial respiratory chain can lead to ROS accumulation and cause mitochondrial stress. Overworked mitochondria gradually decompensate, with reduced efficiency and eventually develop dysfunction, releasing more oxygen molecules. These oxygen molecules further damage mitochondrial DNA, disrupt the respiratory chain again, and exacerbate the OS changes.^[^ ^17^, ^18^, ^19^ ^]^ Both endogenous and environment‐induced exogenous ROS are important factors that influence the nutrition and moisturization on the ocular surface. Moreover, the cornea, TF, LG, conjunctiva, and MG are the principal sites where DED manifests itself. They work in synergy to maintain the normal secretion of tears and the stability of TF, leaving behind a series of traceable OS markers (**Table** **1**).

**Table 1:** Oxidative stress biomarkers in different DED samples.

Sample	Antioxidant		ROS and related enzymes	Lipid peroxide	DNA damage	Protein oxidation	Refs.
	Enzymatic	Nonenzymatic					
Cornea	SODs ↓ / ↑ GPXs ↓ / ↑ CAT ↓ / ↑	AA ↓ GSH ↓ / ↑ GSSG ↑ α‐ tocopherol ↓	ROS ↑ H_2_O_2_ ↑ MPO ↑ O_2_^•−^ ↑ NOX4 ↑	LPO ↑ MDA ↑ 4HNE ↑	8‐OHdG ↑	3‐NT ↑	[4, 18, 25, 27, 28, 29, 30, 31, 38, 40, 70, 82]
Tear film	SODs ↓ GPXs ↓ / ↑	GSH ↓ Ferritin ↓	ROS ↑ H_2_O_2_ ↑ MPO ↑	MDA ↑ 4HNE ↑ HEL – / ↑			[37, 39, 40, 41, 42, 46, 47, 49]
Lacrimal gland	SODs ↓ GPXs ↓	GSH ↓ Ferritin ↓	ROS ↑ O_2_^•−^ ↑	LPO ↑ MDA ↑ 4HNE ↑	8‐OHdG ↑		[32, 49, 51, 54, 61, 64, 65]
Conjunctiva	SODs ↓ GPXs ↓ CAT ↓	GSH ↑ GSSG ↑	ROS ↑ H_2_O_2_ ↑ O_2_^•−^ ↑	4HNE ↑ HEL ↑/	8‐OHdG ↑	3‐NT ↑	[18, 40, 47, 50, 69, 70, 83]
Meibomian gland	SODs ↓ HO‐1 ↓		ROS ↑ NOX4 ↑	4HNE ↑	8‐OHdG ↑		[32, 51, 80]

### 2.1 Cornea

The cornea forms the first line of defense against ROS in the anterior segment, thus demonstrating a strong sensitivity to it. There are numerous enzymatic and non‐enzymatic antioxidants in each layer of the cornea. Among them, the epithelial layer, as a core component of the innate immune system, has the largest variety and content of antioxidants detected so far. It serves as an important repository for superoxide dismutase (SODs), glutathione peroxidases (GPXs), catalase (CAT), glutathione (GSH), α‐tocopherol (α‐T), retinol, lactoferrin, etc.^[^ ^20^ ^]^ The endothelial and stromal layers also contain partial antioxidants such as SODs, CAT, and GSH. As people age, the activity of SODs in corneal endothelial cells decreases, and mitochondrial ROS (mtROS) accumulates. In the later stages of life, the antioxidant function of the ocular surface tissues almost disappears.^[^ ^14^, ^20^, ^21^ ^]^ Therefore, it has been speculated that this is one of the reasons for age‐related DED. Compared with normal individuals, the corneal epithelium of DED patients becomes thinner, with a decreased cell density and abnormal cell arrangement. The microvilli of the superficial epithelium decrease, which is detrimental to the anchoring of the TF. Progenitor cells in the corneal limbus respond to cell renewal through transient amplification to compensate for the losses. But it cannot be maintained for a long time. Ultimately, collagen degradation occurs and the cornea suffers.^[^ ^22^ ^]^ Previously, it was held that this was a consequence of inflammation and THO. But it appears that OS is also involved in it now.

Excessive ROS accumulation was found in the corneas of DED subjects.^[^ ^23^ ^]^ Treatment targeted at mtROS can effectively promote the repair of the corneal epithelium, alleviate the inflammation in the stroma, and relieve dryness.^[^ ^24^ ^]^ In the corneal epithelial cells of DED induced by hypertonicity, the activities of SODs, GPXs, and CAT are significantly lower than those in the control group.^[^ ^25^ ^]^ Based on this, Jingguo, et al. further detected increased expression of 3‐nitrotyrosine (3‐NT), which is a product of protein nitration.^[^ ^18^ ^]^ It is positively correlated with the content of ROS and reactive nitrogen species and thus is regarded as a classic OS damage marker.^[^ ^26^ ^]^ 3‐NT elevation in the cornea implies a mutual promotion between DED and ROS‐induced intracellular protein modification and functional changes. Huaqiong, et al. induced DED mice with excessive tear evaporation using a high‐speed hair dryer. Compared with healthy mice, their corneal cells appeared to have 3‐NT accumulation and apoptosis. Meanwhile, the expression of NOX4, a source of ROS, was increased, and the secretion of matrix metalloproteinase‐9 was activated, which disrupted the tight junctions of the corneal epithelium and damaged the barrier.^[^ ^27^ ^]^ In the models induced by benzalkonium chloride, the levels of GPXs and GSH were also decreased. Their limbal progenitor cells expanded into the periphery, suggesting continuous damage and repair during OS change.^[^ ^4^, ^17^ ^]^ A lot of lipid peroxide (LPO) was detected there.^[^ ^28^ ^]^ Besides, the malonaldehyde (MDA), 4‐hydroxynonenal (4‐HNE) and 8‐hydroxy‐2′‐ deoxyguanosine (8‐OHdG) were validated to significantly increase in the corneas of DED animals induced by dry environment,^[^ ^29^ ^]^ blink inhibition,^[^ ^30^ ^]^ ultraviolet radiation and mechanical damage,^[^ ^31^ ^]^ respectively. It is believed that ROS attacks the polyunsaturated fatty acids of the cell membrane, forming covalent bonds between the LPO and the receptors on the membrane, thus destroying its integrity. MDA and 4‐HNE are lipid peroxidation products of the membrane. They react with deoxynucleosides and damage DNA.^[^ ^32^ ^]^ The persistently high level of 8‐OHdG is the result of DNA repair disorder. The increases in all three imply oxidative damage to the cornea.

Interestingly, Senin, Ivan I found a decrease in SOD, GPX, GSH, ascorbic acid (AA), and α‐T in the cornea,^[^ ^31^ ^]^ while Kazuo, et al. detected an increase in SOD, GPX, and CAT in another experiment.^[^ ^30^ ^]^ These two results are not in conflict. Initially, during low‐intensity OS, the corneal antioxidant system will respond to the changes and be enhanced to protect the ocular surface. Nevertheless, when the ROS exceeds the antioxidant capacity, the balance is disrupted. The cornea degenerates in chronic OS, and the regenerative ability of epithelial cells declines. Eventually, the activities of antioxidants drop to the lowest level.^[^ ^33^ ^]^ Therefore, the trends of the increase both in ROS and OS damage markers in DED are consistent. Certainly, these data also have limitations. Neither cell lines nor animal models can fully replicate the genetic background, physiological characteristics, or metabolism of humans. Hayden, Patrick reconstructed the 3D human corneal epithelium in vitro. In different DED models induced by ultraviolet light, dryness, and chemical agents, the expressions of 32 OS‐related genes showed more than two‐fold up‐regulation or down‐regulation, which was statistically significant. The morphological changes of DED corneal cells were consistent with the amount and duration of ROS accumulation.^[^ ^34^ ^]^ It is without doubt that the corneal damage induced by ROS is the key to DED's occurrence.

### 2.2 Tear and Tear Film

The TF covers the front of the cornea. When eyes open, a stable TF is rebuilt on the ocular surface to achieve deposition and redistribution so as to provide moisture and protection.^[^ ^1^, ^35^ ^]^ Therefore, DED‐related discomfort and foreign body sensation are probably attributed to the TF disorder, excessive evaporation of tears, and too rapid rupture of the TF. Meanwhile, such phenomena will also affect the normal osmotic pressure of tears, in which there are various ions and macromolecular substances. When their osmotic pressure is gradually higher than that of corneal epithelial cells, namely the “THO” in terminology, the corneal barrier is at risk of destruction. Consequently, corneal cells undergo apoptosis, the loss of goblet cells (GCs) occurs, and the ocular microenvironment goes chaotic.^[^ ^36^, ^37^ ^]^

THO is a core mechanism of DED. In addition to inducing immune inflammation, it can cause severe and persistent OS damage on the surface. Myeloperoxidase (MPO), a peroxidase mainly expressed in neutrophils, can link inflammation and OS, and catalyze oxidation reactions to generate plentiful ROS.^[^ ^38^ ^]^ Their activity in the tear samples of DED patients was enhanced,^[^ ^39^ ^]^ and ROS level was increased simultaneously, which triggered a higher expression of GPXs.^[^ ^40^, ^41^ ^]^ Additionally, the lactoferrin in the TF of severe DED patients was significantly reduced.^[^ ^42^ ^]^ Generally speaking, only a very low level of free iron exists on the ocular surface.^[^ ^20^ ^]^ The free iron increase will prompt the generation of the more reactive OH^•^ from H_2_O_2_, leading to further damage. Lactoferrin in tears can act as an iron chelator to inhibit this.^[^ ^43^ ^]^ Therefore, it is considered the main antioxidant in the TF. Supplementing lactoferrin in the diet reduces OS and inflammation in DED patients, and thus relieves dryness.^[^ ^44^ ^]^ Similarly, lactoferrin eye drops also show good efficacy in animal models.^[^ ^45^ ^]^ In the tears of medium and severe DED patients, the levels of MDA, 4‐HNE,^[^ ^46^ ^]^ and hexyl lysine (HEL)^[^ ^47^ ^]^ were significantly elevated. MDA and 4‐HNE are markers of advanced LPO, while HEL is a decomposition product of early lipid peroxidation. It is also a messenger of oxygen free radicals and has cytotoxicity, which indicates extensive lipid oxidative damage.^[^ ^48^ ^]^ Besides, DED mice induced by sleep deprivation had tears of a high level of H_2_O_2_, along with low expressions of GSH and GPXs.^[^ ^49^ ^]^ SOD1‐knockout mice showed a deterioration of the TF function, which was considered to mimic age‐related DED.^[^ ^50^, ^51^ ^]^ When excessive ROS were removed, the quality and quantity of tears and the stability of the TF were all obviously improved.^[^ ^51^ ^]^

**Figure 2:**
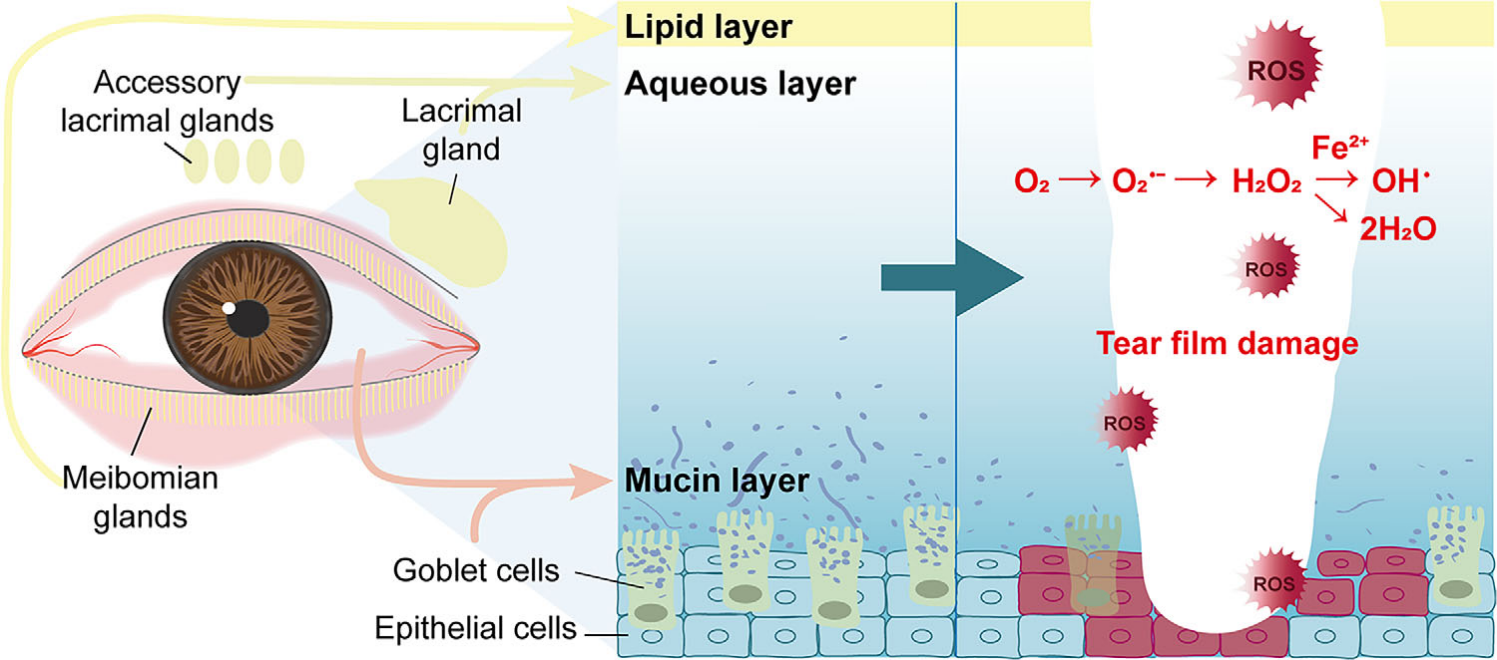
Tear film composition and the corresponding sources.

What merits emphasis is that the origin of these changes and the key to reversing the oxidative damage do not lie solely within the tears themselves. A widely held view is that the TF can be divided into three layers: the lipid layer externally, the aqueous layer in the middle, and the mucin layer adjacent to the cornea (**Figure** **2**).^[^ ^52^ ^]^ Any pathological alterations in one of these components will result in diminished lubrication and increased friction on the ocular surface. The lipid layer is mostly derived from the secretions produced by the MG, the aqueous layer is mainly secreted by the epithelial cells of the LG, and the mucins are principally generated from the conjunctival epithelial GCs, corneal epithelial cells, and LG acinar cells.^[^ ^53^ ^]^ To sum up, a stable TF is jointly established by different tissues.

### 2.3 Lacrimal Gland (LG)

The main and accessory LGs contribute the most to the composition of the TF, specifically, the aqueous layer. The decrease in GPX2, GPX3, GPX4, and lactoferrin in tears was associated with the reduced activity of GPXs and lactoferrin in the LGs.^[^ ^49^ ^]^ Unlike the structures that are directly affected by environmental impacts, LGs are relatively less sensitive. But they are highly susceptible to aging. On the one hand, whether it is age‐related senescence of LGs,^[^ ^54^ ^]^ or senescence induced by sleep deprivation,^[^ ^49^ ^]^ dysbiosis,^[^ ^55^ ^]^ long‐term smoking,^[^ ^56^ ^]^ and high‐fat diet,^[^ ^57^ ^]^ there is a ROS accumulation which impairs the secretory quality, cell size, and transcriptome level of the glands. On the other hand, after aging, even in a state of balance, chronic oxidative damage still occurs in aerobic cells.^[^ ^54^, ^55^ ^]^ The activity of antioxidants in the LGs decreases, and the unremoved ROS continuously influences chromosomes and mitochondria and exacerbates cellular senescence through its impact on epigenetic modifications.^[^ ^32^, ^58^ ^]^ This interconnected pathological process persistently damages the acinar and duct cells, leading to aqueous‐deficient DED.

Healthy acinar cells of LGs are homogeneous in size, arranged neatly, and possess normal morphology. In contrast, the acinar cells of DED patients display signs of atrophy, accompanied by inflammation, periductal fibrosis, and cystic ductal dilation.^[^ ^59^ ^]^ OS can induce such pathology. Tsubota, et al. developed a novel mev‐1 transgenic DED model.^[^ ^60^, ^61^ ^]^ The mev‐1 level impacts the mitochondrial electron transport chain within the LGs, and elevates the levels of O_2_^−^and 8‐OHdG. As a result, the acinar cells finally atrophy, along with multifocal inflammation and fibrosis, just like DED patients. OBATA et al. observed obvious nuclear fragmentation and vacuolar changes in the LGs of SOD1‐knockout mice.^[^ ^59^ ^]^ SOD1 deficiency leads to an augmentation in the content of 4‐HNE and DNA damage. The mitochondria become swollen, and disoriented, and exhibit disordered cristae under the assault of ROS. Consequently, they are unable to function properly to supply power for the secretory function of the LGs. Additionally, the lipofuscin piling up might constitute new evidence for ROS‐induced damage.^[^ ^37^, ^54^ ^]^ Lipofuscin is an insoluble lysosomal polymer. Highly reactive aldehydes are generated during lipid peroxidation, which crosslinks with oxidized proteins. Lipofuscin is mainly composed of these crosslinked protein residues.^[^ ^62^ ^]^ Therefore, OS promotes lipofuscin production, and intractable lipofuscin further aggravates cell aging and induces ROS accumulation.^[^ ^37^ ^]^ In the LGs of age‐related DED, excessive lipofuscin, LPO, and decreased levels of GSH coexist.^[^ ^54^ ^]^

Besides, ROS can also damage the autonomic nerves that innervate the LGs and the myelin sheaths of the afferent sensory nerves, resulting in a reduction in the signals transmitted and insufficient tear secretion.^[^ ^63^ ^]^ Eldien et al. have confirmed that calorie restriction inhibits the loss of parasympathetic, sympathetic, and sensory nerves in the fibrotic area of the LG and improves the atrophy of the acinar and duct cells. It involves promoting the expression of antioxidants and reducing the MDA.^[^ ^64^ ^]^ Meanwhile, such an intervention restrains the generation of 8‐OHdG and 4‐HNE, and protects the integrity of mitochondria.^[^ ^65^ ^]^ Takaaki, et al. found that inducing SOD1 and SOD2 can inhibit the accumulation of secretory vesicles in the acinar epithelial cells of the LGs.^[^ ^51^ ^]^ In addition, Roth et al. have verified that scavenging ROS in the LGs helps to preserve the vitality of LG‐derived mesenchymal stem cells, enabling them to have higher proliferation, differentiation, and regeneration capabilities.^[^ ^66^ ^]^

### 2.4 Conjunctiva

The conjunctiva houses accessory LGs. Apart from this, the conjunctival GCs secrete high‐molecular‐weight and highly glycosylated proteins termed mucins. Mucins are mainly of two types: membrane‐associated mucins (MAM) and secretory mucins (SM). On the microvilli covering the conjunctival and corneal epithelial cells, there is a polysaccharide–protein complex composed of glycoproteins, proteoglycans, and glycolipids, namely the glycocalyx. MAM, represented by MUC1, MUC16, and MUC4, contributes to the formation of the glycocalyx barrier. It aids in fending off the encroachment of pathogenic microorganisms and transforms the ocular surface from hydrophobic to hydrophilic, thereby diminishing friction and augmenting moisture.^[^ ^52^ ^]^ SM, exemplified by MUC5AC, takes part in the constitution of the mucin layer within the TF, serving a “water retention” function. This allows the TF to adhere to the ocular surface more stably.^[^ ^67^ ^]^ Therefore, if too little mucin is secreted, the ocular surface will tend to be hydrophobic.

There is always a loss of GCs and a low expression of MUC5AC in the conjunctiva of patients with moderate and severe DED.^[^ ^68^ ^]^ Meanwhile, Chen, et al. found that both ROS and inflammatory factors increased in this region.^[^ ^40^ ^]^ Cejková et al. detected that SODs, GPXs, and CAT in the conjunctiva decreased significantly as the severity of ocular dryness intensified.^[^ ^69^ ^]^ Additionally, accumulations of 4‐HNE and HEL were observed, signifying excessive oxidative damage.^[^ ^47^ ^]^ In the conjunctiva of SOD1‐knockout mice, the epithelium flattened, the microvilli became blunted, and squamous metaplasia of the conjunctiva occurred, along with an increase in the levels of 8‐OHdG and a decrease in the number of GCs.^[^ ^50^ ^]^ Compared with the cornea, which enjoys an immune‐privileged condition for its unique avascular structure, the conjunctiva exhibits greater susceptibility to OS. It is more reactive to phototoxic stress because of its epithelium replete with highly interconnected immunocompetent cells. Irradiated by blue light, human conjunctival cells are prone to mitochondrial membrane potential changes and increased levels of O_2_^−^ and H_2_O_2_.^[^ ^70^ ^]^ GSH is activated in response to ROS accumulation. The continuous expression of oxidized GSH (GSSG) and the decline in GPX1 activity once again suggest an imbalance.^[^ ^70^ ^]^ In hypertonic‐induced conjunctival cells, there are also high levels of 3‐NT indicating a strong OS state.^[^ ^18^ ^]^

Regrettably, in the conjunctiva of animals with MUC1^[^ ^71^ ^]^ and MUC16^[^ ^72^ ^]^ gene knockouts, only an upsurge in inflammatory factors has been noted. There is currently no report regarding OS‐related indicators. Hence, direct evidence validating the decrease in mucin and the existence of OS is currently in short supply. But many studies have shown that antioxidants can scavenge ROS, reduce the loss of GCs, and target mucin secretion.^[^ ^10^, ^73^ ^]^ There is no doubt that improving the OS in the unhealthy conjunctiva is of great benefit to the DED treatment.

### 2.5 Meibomian Glands (MG)

The MG, being the largest sebaceous gland in the body, lies within the tarsal plate of the eyelid and is capable of secreting meibum, which contains abundant lipids. Nonpolar hydrophobic and polar amphiphilic lipids overlay on the ocular surface, constituting the primary source of the lipid layer. The lipid layer mitigates the surface tension of the TF, promotes the spread of tears during blinking, and decelerates its rate of evaporation. On the other hand, the meibum stored at the edge of the eyelid can maintain the hydrophobic state of the surrounding skin and prevent the overflow of tears.^[^ ^74^ ^]^ Therefore, MG dysfunction and lipid disorders are risk factors for evaporative DED. On average, healthy people have 31 glands in the upper eyelid and 26 glands in the lower eyelid, while the number of functional glands in DED patients is significantly reduced.^[^ ^75^, ^76^ ^]^ The increased ROS may be involved in the pathology. First, the lipid layer is the outermost structure which is extremely vulnerable to environmental ROS attacks. Second, the unsaturated fatty acids in lipids are prone to oxidation reactions to generate harmful LPO, which amplifies OS and inflammation and continuously damage tissue cells. In the DED treatment, high levels of SOD1 and GPXs in the MG play a crucial role in interrupting the OS‐inflammation cycle.^[^ ^77^ ^]^

Tsubota, et al. discovered a significant increase in 4‐HNE and 8‐OHdG in the MG of SOD1–knockout mice, accompanied by increased peripheral inflammation and glandular fibrosis.^[^ ^78^ ^]^ It was in line with the pathological research results of FUT1‐knockout MG dysfunction mice by Yoon, C H' s team.^[^ ^79^ ^]^ In another experiment, the increases in NOX4 and ROS of MG were positively correlated with the degree of acinar shrinkage and the destruction of the acinar basement membrane. The expressions of heme oxygenase‐1 (HO‐1) and SOD2 were obviously downregulated.^[^ ^80^ ^]^ HO‐1 is an important antioxidase that is responsible for catalyzing the catabolism of heme into ferrous iron, carbon monoxide, and biliverdin. Biliverdin and its reduced form, bilirubin, can resist peroxides, peroxynitrite, hydroxyl, and superoxide radicals and scavenge ROS. Meanwhile, heme degradation can help block the pro‐oxidative process.^[^ ^81^ ^]^ Multiple data have confirmed that the imbalance of the antioxidant system in the MG can lead to abnormal differentiation of acinar cells and keratinization of duct cells, ultimately resulting in duct obstruction. As a consequence, lipids accumulate due to the inability to be discharged smoothly, leading to DED. Targeted modulation of OS in MG will emerge as a highly promising research direction. However, the specific molecular mechanisms in the MG of DED patients still require validation with large‐scale clinical samples.

## 3 Inflammation and Oxidative Stress

Inflammatory cell infiltration is a prominent pathological feature of DED. The continuously vigilant innate immunity and abnormal adaptive immunity are both involved in DED.^[^ ^84^ ^]^ It has been reported that in addition to directly damaging the ocular surface system, the ROS itself and oxidative damage products can also act as mediators to initiate the nuclear import and transcription process of nuclear factor kappa‐B (NF‐κB), promoting the expression of genes related to inflammatory immunity.^[^ ^70^ ^]^

There is a significant increase in interleukin‐1β (IL‐1β), IL‐8, IL‐6, and tumor necrosis factor‐α (TNF‐α) in the corneas, LGs, and conjunctivas of DED patients.^[^ ^1^ ^]^ These factors promote the maturation of antigen‐presenting cells (APCs). After activation, APCs migrate to the regional draining lymph nodes and induce naive T cells (Th0) to differentiate into T helper cell 1 (Th1) which secretes interferon‐γ (IFN‐γ), and Th17 which secretes IL‐17.^[^ ^1^ ^]^ IFN‐γ can regulate the expression of chemokines and cell adhesion factors to recruit more inflammatory cells on the ocular surface. Meanwhile, IL‐17 can induce the expression of matrix metalloproteinases (MMPs), such as MMP‐1, MMP‐3, MMP‐9, and MMP‐13, together with pro‐inflammatory factors such as IL‐1β, IL‐8, and TNF‐α, destroying the tight junctions between corneal epithelial cells and leading to barrier damage.^[^ ^7^, ^85^ ^]^ The NF‐κB transcription pathway activated by ROS is considered one of the potential mechanisms of DED. In addition, the damage of ROS to cell membranes and organelles such as mitochondria may lead to the release of damage‐associated molecular patterns (DAMPs), activate inflammatory surface receptors, initiate the assembly and activation of nucleotide‐binding oligomerization domain‐like receptor protein 3 (NLRP3). It promotes the maturation and release of the IL‐1β precursor. Antioxidants can interrupt the inflammation mediated by the NLRP3 pathway.^[^ ^85^ ^]^

During inflammation reactions, the activated leukocytes exposed on the ocular surface may produce a large amount of O_2_^•−^, H_2_O_2_, and even the more active OH^•^.^[^ ^86^ ^]^ Thus, sometimes ROS is a weapon for immune cells to attack normal structures. Inflammatory infiltration can also exacerbate oxidative damage to the ocular surface and upregulate the expression of cell surface ROS‐generating enzymes, such as NOX. Breaking the vicious cycle of OS inflammation in the eyes of DED patients will be a more promising treatment option than simple anti‐inflammatory treatment.

## 4 Antioxidant Therapy for DED

### 4.1 SKQ1

SKQ1 is the first mitochondria‐targeted antioxidant applied in a clinical setting and was approved for sale as a prescription drug in Russia in 2011. Compared with natural antioxidants, SKQ1 exhibits an enhanced mitochondrial penetration ability, which can bypass the plasma membrane and enter mitochondria to neutralize excessive ROS in situ.^[^ ^87^ ^]^ In the Phase 3 Clinical Trial of SKQ1 eye drops, it can effectively improve low tear secretion, short TF break‐up time, and corneal epithelial defect in DED patients, with few adverse reactions.^[^ ^88^ ^]^ Senin et al. believe that SKQ1 not only scavenges mtROS, but also enhances the intrinsic antioxidant defense system of the cornea, promotes corneal re‐epithelialization and stromal remodeling, and reduces the incidence of subsequent inflammation.^[^ ^31^ ^]^ Besides, Lin, Sen's team further developed a kind of SKQ1 nanoparticles by charge‐driven self‐assembly. The innovative SKQ1 nanoparticles have higher bioavailability and biocompatibility owing to the integration of poly (ethylene glycol)‐poly (glutamic acid) (PEG – PGA) copolymer. In contrast to free SKQ1, SKQ1 nanoparticles demonstrate a prolonged duration of action and a more potent ROS scavenging capacity. It protects the mitochondrial DNA from oxidative damage, and suppresses the expression levels of NLRP3, Caspase‐1 and IL‐1β.^[^ ^88^ ^]^

### 4.2 Rebamipide

Rebamipide is initially a potent antioxidant for treating gastric ulcers. It can activate the nuclear factor erythroid‐derived 2‐like 2 (Nrf2) / HO‐1 pathway, scavenge oxygen free radicals, promote mucin secretion, and protect the gastric mucosa.^[^ ^89^ ^]^ Taking advantage of the common characteristic that the stability of both gastric mucus and TF depends on the presence of the mucin barrier, Japan took the lead in developing rebamipide ophthalmic suspension for DED treatment and launched it locally in 2012. These eye drops also exhibits excellent mucin‐secreting activity on the ocular surface. It promotes the gene expressions of MUC1, MUC4, MUC16, and MUC5AC, increase the count of conjunctival GCs, repairs the corneal epithelium, and improve the damage to microvilli.^[^ ^90^ ^]^ Compared with other clinical DED drugs, rebamipide ophthalmic suspension contains no preservatives, which reduces cytotoxicity and decreases the incidence of OS on the ocular surface. Even more importantly, it is a product that targets mucin rather than simply increasing the volume of tears. The pity is that the Phase 3 Clinical Trial results of Rebamipide showed 9.7% of the DED patients developed taste disorders after treatment.^[^ ^91^ ^]^ Therefore, the adverse effects of this eye drop remain to be investigated.

### 4.3 Mitoquinone

Mitoquinone is a mitochondria‐targeted antioxidant developed by a New Zealand team that can block H_2_O_2_. Essentially, it consists of the coenzyme Q10 loaded with the lipophilic cation triphenylphosphine. The interior of the mitochondrial membrane has a negative potential. The coupling of triphenylphosphine can assist coenzyme Q10 to be attracted to the inner mitochondrial membrane, increase its antioxidant properties without interfering with the normal mitochondrial respiratory chain, and prevent oxidative damage.^[^ ^92^ ^]^ Lin, et al. prepared Mitoquinone eye drops by charge‐driven self‐assembly. 0.1 mm Mitoquinone nanoparticles alleviated the ROS accumulation caused by THO and significantly inhibited the expressions of IL‐1β and NLRP3.^[^ ^93^ ^]^ Meanwhile, such cationic nanoparticles are helpful for enhancing the adhesion of drugs on the negatively charged cornea. It can provide inspiration for the development of corneal medications for DED.

### 4.4 Elamipretide

Elamipretide is a cell‐penetrating peptide with an aromatic‐cationic structure. Just like mitoquinone, it is also a mitochondrially targeted antioxidant. But the uptake of elamipretide does not depend on the transmembrane potential and will not cause mitochondrial depolarization. It can stabilize the integrity of mitochondrial cristae and the electron transport chain during OS changes, so as to reduce ROS production.^[^ ^94^ ^]^ Xia, Y's team prepared eye drops by simultaneously loading elamipretide and anti‐inflammatory ingredient, insulin, into multilamellar liposomes. Eventually, the dual‐action medications synergistically restore mitochondrial function in the cornea and conjunctiva of DED. The mechanism involves activating GPX4, scavenging ROS, and reducing glutamate levels. It leads to the consumption of substrates in the tricarboxylic acid cycle and reduces fumarate.^[^ ^17^ ^]^

### 4.5 SOD and SOD Mimetic

As the mainly antioxidase, SOD catalyzes the conversion of O_2_^•−^ to H_2_O_2_ through the gain and loss of electrons of the metal ions in the active center, paving the way for the subsequent decomposition of H_2_O_2_ into water and oxygen.^[^ ^20^ ^]^ The activities of SOD in the cornea, conjunctiva, TF, LGs, and MG in DED patients are all significantly decreased. Therefore, SOD1‐knockout mice have become the most common models in DED research. Directly supplementing SOD provides a possibility for treatment. However, high‐molecular‐weight SOD is difficult to be absorbed in the gastrointestinal tract and the decomposed SOD is inactive. Yoon et al. designed a new oral administration of bacillus‐derived SOD. It can increase the expression of autologous SOD2, reduce ROS and MDA, and alleviate the damage to corneal epithelial cells and conjunctival GCs.^[^ ^23^ ^]^ Compared with topical administration, this approach avoids the potential side effects of frequent use of eye drops, but the specific bioavailability still requires more experimental data.

2,2,6,6‐Tetramethylpiperidin‐1‐oxyl (TEMPO) is a small‐molecule SOD mimetic. It can more easily penetrate cell membranes, capture free radicals, and scavenge O_2_^−^.^[^ ^13^ ^]^ Han, et al. designed a cationic micelle eye drop loaded with TEMPO. Eventually, 100 µg mL^−1^ TEMPO eliminated 72.9% of H_2_O_2_, inhibited the p38 MAPK pathway, and regulated the expressions of downstream cytokines such as MMP‐9 and IL‐1β.^[^ ^7^ ^]^ Corneal barrier damage caused by the ROS was successfully repaired. Nevertheless, according to another report, TEMPO can also act as a catalyst to promote the oxidation process under certain circumstances.^[^ ^95^ ^]^ Therefore, whether the antioxidant property of TEMPO in DED treatment is stable remains to be considered.

### 4.6 Lactoferrin

Lactoferrin accounts for 25% of all tear proteins. Its stable and reversible iron‐binding ability can handle free iron and OH^•^.^[^ ^43^ ^]^ The content of lactoferrin in tears of DED patients is halved.^[^ ^96^ ^]^ Supplementing Lactoferrin can restore the integrity of the corneal epithelium in different DED animal models.^[^ ^97^ ^]^ Lactoferrin eye drops encapsulated by liposomes are effective in relieving corneal OS, and they can increase tear secretion.^[^ ^45^ ^]^ Lecchi, et al. experimented with the possibility of contact lenses (CLs) loaded with lactoferrin, which continuously reduced H_2_O_2_ within 24 h in DED models.^[^ ^98^ ^]^ However, the specific efficacy and adverse reactions in vivo have not been reported yet. Oral administration of lactoferrin can effectively protect the corneas of DED mice from ROS accumulation and reduce 8‐OHdG.^[^ ^99^ ^]^ Similarly, lactoferrin enteric capsules at a dose of 270 mg per day also show good curative effect in DED patients, which promotes the GCs secretion and prolongs the TF break‐up time.^[^ ^100^ ^]^ What should be noted is that excessive lactoferrin supplementation may lead to an overload of iron transport, affect the absorption of other nutrients, and impose a burden on the gastrointestinal tract.

### 4.7 Nanoenzymes

Nanozymes are metal‐based nanoparticles and artificial biomimetic materials with enzyme‐catalytic features. They mimic natural antioxidase and have cost‐effectiveness and high stability compared to natural enzymes and small‐molecule antioxidants. Also, they can act both as drugs and as nanocarriers for drug loading. The development of DED eye drops with nanozyme formulations has attracted great attention recently.

#### 4.7.1 Cerium Oxide

Cerium oxide nanoparticles (CeNPs) are one of the most frequently reported nanozymes. The reversible Ce^3+^/Ce^4+^ redox couple enables them to mimic SODs and CAT.^[^ ^101^ ^]^ Due to the special crystal structures, CeNPs have very low solubility, resulting in limited bioavailability in vivo. Based on this, Han, Zongchao's team designed a CeNPs eye drop encapsulated by ethylene glycol chitosan. With the support of the hydrosoluble carrier, it significantly improved the conjunctival GCs loss caused by OS and repaired the corneal epithelium.^[^ ^73^ ^]^ Lin, et al. used the positively charged branched polyethyleneimine ‐graft‐polyethylene glycol (bPEI‐g‐PEG) as a carrier to prepare CeNPs@bPEI‐g‐PEG eye drops. The solubility of CeNPs and the drug uptake capacity of the cell membrane were enhanced. By leveraging the nanocarrier, CeNPs achieved precise delivery and controlled – release on the ocular surface. It reduced the levels of O_2_^−^, OH^•^, and H_2_O_2_, restored cell vitality, and improved the OS damage.^[^ ^102^ ^]^ Additionally, there are also CeNPs administration therapies encapsulated by exosomes, metal–organic frames (MOFs), and CLs. Exosomes are a subgroup of extracellular vesicles and can transfer CeNPs into the body via endocytosis or the direct fusion of vesicles with cell membranes. Their low immunogenicity can prevent CeNPs from being recognized or cleared by the immune system.^[^ ^103^ ^]^ Tao, et al. have developed MSCExo – CeNPs eye drops by in situ crystallization. The inherent anti‐inflammatory property of exosomes can enhance the antioxidative effect of CeNPs, synergistically treat DED, and improve efficacy.^[^ ^104^ ^]^ MOFs are a class of porous hybrid materials constructed by the coordination of metal ions and organic bridging ligands. The adjustable pore size enables them to be personalized and designed according to requirements, achieving efficient loading and precise release of drugs. It is currently believed that the size of CeNPs is a key factor in inhibiting OS. Therefore, Huang, et al. customized three different sizes of CeNPs‐MOF eye drops for DED treatment. Among them, the CeNPs‐MOF 3 with an ultra‐small particle size (2 nm) had the best scavenging effects on O_2_^−^ and H_2_O_2_. It well maintains the normal morphology and functions of cornea and conjunctiva, and promotes tear secretion.^[^ ^105^ ^]^ Kim, et al. embedded CeNPs into CLs to enhance convenience. The CLs could be worn for a period of time. Consequently, CeNPs continuously contact the TF, cornea, and conjunctiva through the platform of CLs, producing a stronger effect of reducing tear evaporation and prolonging the drug retention time. The antioxidase in vivo was activated to reduce ROS, with few side‐effects.^[^ ^106^ ^]^

Other than this, CeNPs with high specific surface area are also excellent materials of carriers. Lin, Quankui's team designed Ce@PBD eye drops, which encapsulated dikophoxone sodium (DQS) with CeNPs to re‐establish the oxidative‐antioxidant balance.^[^ ^3^ ^]^ Compared with DED mice treated with DQS alone, those treated with Ce@PBD had a more complete corneal structure and a significant increase in mucin secretion. Their tear volume was closer to the normal level. It was related to the inhibition of ROS accumulation, the down‐regulation of the Toll‐like receptor 4/myeloid differentiation factor 88/NF‐κB (TLR4/MyD88/NF‐κB) pathway, and the reduction of IL‐1β and TNF‐α expression.^[^ ^3^ ^]^ Han, and Haijie developed Cs@P/CeO2 dual‐function eye drops, which encapsulated CsA within CeNPs.^[^ ^4^ ^]^ It led to higher GPX4 expression in the corneal and stimulated secretion of conjunctival GCs. The vicious cycle of OS‐inflammation was blocked, resulting in a better treatment effect than using CsA alone.

#### 4.7.2 Iron‐Based Nanozymes

Ferrum (Fe) is an important transition element in the human body. Prussian blue nanoparticles (PBNPs), namely the iron ferrocyanide, have peroxidase‐like activity. The iron ions (Fe^3^⁺ and Fe^2^⁺) inside it can participate in the electron transfer process and catalyze the conversion of H_2_O_2_ into water and oxygen.^[^ ^107^ ^]^ Du, Yan's team developed CLs loaded with PBNPs. To better simulate the ocular surface where tears rapidly update in humans, they simultaneously conducted artificial tear buffer flushing in two different DED animals induced by ultraviolet and H_2_O_2_. In this condition, the coverage of PBNPs‐CLs could still produce real‐time ROS scavenging for two consecutive months, including O_2_^−^, H_2_O_2_, and OH^•^. The application of PBNPs‐CLs compensated for the loss of corneal epithelial and stromal cells, and inhibit the expression of TNF‐α and IL‐1β both in vivo and in vitro, while increasing the level of IL‐10.^[^ ^21^ ^]^

Li, et al. embedded monatomic Fe into the hydrogel containing borate bonds to prepare iron‐based nanozyme eye drops. The dynamic covalent bonds of borate bonds improved the solubility and dispersibility of the nanozyme. Meanwhile, the characteristics of the hydrogel enhanced the adhesion of the drug and prolonged its action time. As reported, the eye drops successfully increased the expression of SOD1, CAT, and GPX1, alleviated oxidative damage, and reduced the expressions of IL – 1β and IL‐6.^[^ ^108^ ^]^ Besides, on the basis of single atoms, Li, et al. also attempted to simultaneously embed Fe and Manganese (Mn) into nitrogen‐doped carbon materials and modify them with hydrophilic polymers to prepare a brand‐new antioxidant dual‐atom nanozyme (DAN) eye drops. Finally, DAN performed excellently in scavenging mtROS in the cornea and conjunctiva and promoting tear secretion. The mechanism involved the regulation of the ROS/NLRP3/IL‐1β pathway, for which the activities of SOD1, CAT, GPX1, and HO‐1 were restored, the activation of NLRP3 inflammasome was inhibited, and the expressions of pro‐inflammatory cytokines, IL‐1β and IL‐18, were reduced.^[^ ^25^ ^]^

#### 4.7.3 Manganese‐Porphyrin

Mn is the metal ion at the active centers of Mn‐SOD and Mn‐CAT. An appropriate amount of Mn^2+^ can significantly increase the levels of SOD and peroxidase.^[^ ^109^ ^]^ Manganese‐porphyrin is a compound formed by the coordination of Mn with the center of the porphyrin ring. It is helpful for scavenging O_2_^−^, H_2_O_2_, peroxynitrite, and lipid peroxyl radicals, and has a wider range of antioxidant effects. Kaja, et al. have demonstrated that the manganese‐porphyrin eye drops can effectively improve the OS environment of the corneas and LGs of DED mice, reduce the expression of 8‐OHdG, and promote tear secretion and corneal repair.^[^ ^5^ ^]^

#### 4.7.4 Selenium‐Based Nanozymes

Essentially, GPX is a regulatory storage form of selenium (Se) in the body, and Se is the metal ion at the active center of GPX. Copper‐Se nanozymes (Cu2−xSeNPs) can mimic GPX and SODs. Koole's team prepared Cu2−xSeNPs@AF127 hydrogel with Pluronic F127 loaded with drugs, which can regulate the Nrf2 and p38 MAPK pathways to scavenge H_2_O_2_ and OH^•^, reducing corneal inflammation.^[^ ^110^ ^]^ Pluronic F127 provides Cu2−xSeNPs eye drops with thermo‐sensitive gelation properties, which helps it to become sticky instantly when in contact with eyes. This protocol contributes to prolonging the action time, reducing the frequency of medication and alleviating drug toxicity. Additionally, Li, et al. prepared ROS – responsive micelles from Se to encapsulate CsA and achieved dual‐target treatment of antioxidant and anti‐inflammatory in DED, which was verified to be beneficial for the repair of the corneal barrier.^[^ ^27^ ^]^

### 4.8 Graphene Quantum Dots (GQDs)

GQDs are 0D carbon nanomaterials less than 100 nm in size, displaying excellent antioxidant properties. Qi, et al. prepared nitrogen‐doped GQD (N – GQDs) eye drops, which both scavenged ROS and activated the Nrf2 signal transduction, thereby enhancing the total antioxidant level.^[^ ^111^ ^]^ Generally speaking, Nrf2 is a key regulatory factor of the endogenous antioxidant system. It binds to Kelch‐like ECH‐associated protein 1 (Keap1), a negative regulatory factor in the cytoplasm, and remains in an inactive state. When cells are exposed to OS, the cysteine residues of Keap1 are modified, leading to a conformational change. As a result, Keap1 cannot effectively mediate the ubiquitination and degradation of Nrf2. Subsequently, Nrf2 dissociates from Keap1, accumulates within the cell, and then translocates to the nucleus. Nrf2 binds to the antioxidant response element (ARE) in the nucleus, thereby promoting the expression of phase II antioxidant enzymes such as HO‐1. The N – GQDs eye drops can obviously increase Nrf2 expression and reduce Keap1.^[^ ^111^ ^]^ However, another report showed that GQD‐generated ROS under blue light accelerated the oxidation of non‐enzymatic antioxidants and the LPO process.^[^ ^112^ ^]^ How does light affect GQD? How to find the balance between antioxidant and pro‐oxidant to stabilize the efficacy of GQD in DED? These are all the questions that need to be answered.

### 4.9 Tetrahedral Frame of Nucleic Acids (tFNA)

tFNA is a functionalized nanoparticle constructed through DNA origami. It features a spatial tetrahedral structure, with each face containing three single‐stranded DNAs, and can directly enter cells without the aid of carriers or drugs. Compared with other products, tFNA has a high output. Its excellent bioactivity and drug‐delivery properties have made it one of the 3D nucleic acid materials that have attracted much attention recently. But it is less involved in ophthalmology, and there have been only tFNA‐based drug‐making studies in the treatment of DED and glaucoma.

tFNA has a natural ROS‐scavenging ability that is independent of sequence and structure. Moreover, it can provide substrate competition for oxidation reaction after being endocytosed, indirectly relieving the OS.^[^ ^113^ ^]^ Liu, Zuguo ’s team found that the corneal and conjunctival mtROS of DED mice injected with tFNA solution (250 nm), were significantly reduced, and the activities of SOD1 and SOD2 were increased, while 3‐NT was decreased. Meanwhile, the cGAS‐STING pathway was inhibited. The expressions of STING, p‐TBK1 and p‐IRF3 were reduced, thus inhibiting the nuclear translocation of NF‐κB and reducing the secretion of MMP‐9 and IL‐6.^[^ ^18^ ^]^ Therefore, it is currently regarded as a potential candidate for DED treatment. Additionally, tFNA offers plentiful modification sites. Drugs can either be modified in the single‐stranded DNA via base pairing or directly encapsulated within the spatial structure to accomplish targeted drug delivery. It is highly likely that the combination of tFNAs with other antioxidants could furnish more stable, powerful, and long‐term therapy. But tFNAs can only load small molecules such as polypeptides because of the size limitation. Upgrading the technology and constructing a more ideal DNA nanostructure, will become the most breakthrough part of the DED antioxidant strategy.

### 4.10 Monomers of Traditional Chinese Medicine

#### 4.10.1 Flavanoids

Xanthohumol is a natural flavonoid compound extracted from lupus. Due to the mercaptan structure, it can stimulate the dissociation of Nrf2 and Keap1 and then the binding with ARE, promoting the expression of HO‐1.^[^ ^114^ ^]^ Based on the poor water‐solubility of xanthohumol, Kaja, et al. encapsulated it with PLGA to prepare antioxidant eye drops.^[^ ^29^ ^]^ As the results showed, it significantly reduced the oxidative damage to the cornea and LGs of DED animals. Compared with the empty PLGA group, the content of 8‐OHdG was significantly reduced.

Catechin has the typical structure of flavonoid compounds and is also a natural polyphenol extracted from dietary sources such as green tea. The phenolic hydroxyl groups can provide hydrogen atoms, endowing the drug with strong antioxidant properties. Catechin can scavenge ROS and chelate metal ions, inhibit the expression of aldehydrase, and induce the production of antioxidase.^[^ ^115^ ^]^ Chih‐Ching, et al. developed eye drops with catechin‐capped gold nanoparticles as the carrier and loaded with amfenac. Eventually, this drug successfully scavenged the ROS in the cornea and conjunctiva of DED rabbits downregulated the NF‐κB pathway, and promoted the expression of MUC5AC.^[^ ^10^ ^]^

#### 4.10.2 Phenolic Acid

Rosmarinic acid (RosA), a water‐soluble phenolic acid compound sourced from rosemary, owes the natural antioxidant effect for its o‐dihydroxyphenyl and phenolic hydroxyl groups. It directly neutralizes oxygen free radicals and enhances the human antioxidant machinery. Haijie, et al. used gelatin to co‐encapsulate RosA and DQS, preparing antioxidant and anti‐inflammatory gel. This ophthalmic gel showed good efficacy both in animal models with insufficient tear secretion and excessive evaporation. Meanwhile, compared with the group treated with DQS alone, the addition of RosA immediately scavenged ROS in the early stage and inhibited inflammation. The expression of MUC1 was increased, while IL‐1β and MMP‐9 related to corneal barrier destruction were decreased in the drug combination group.^[^ ^116^ ^]^

Gallic acid represents a phenolic acid compound that is ubiquitously present in a diverse array of fruits, vegetables, and nuts. According to reported findings, 5 mg mL^−1^ gallic acid eye drops can upregulate the expressions of HO‐1 and NAD(P)H:quinone oxidoreductase 1 (NQO1), and rehabilitate the morphological integrity of the cornea and conjunctiva in DED mice.^[^ ^117^ ^]^ NQO1 functions by promoting the excretion of quinone through a single‐step two‐electron reduction reaction. This action not only blocks the generation of ROS from semi‐hydroquinone during the redox cycle but also sustains the reduced states of ubiquinone and α‐T quinone, thereby safeguarding endogenous antioxidants.^[^ ^118^ ^]^ This restorative mechanism hinges on the upregulation of the Nrf2 pathway.^[^ ^117^ ^]^

#### 4.10.3 Other Compounds

Salidroside is a phenylethanoside compound with resistance to oxidation. Therefore, Kai et al. prepared salidroside eye drops to alleviate the corneal epithelial damage and low tear secretion caused by OS.^[^ ^119^ ^]^ The efficacy might be related to the activation of the AMPK‐Sirt1 pathway and the acceleration of the nuclear translocation of Nrf2. The increased expression of HO‐1 and NQO1 compensated for the imbalance of antioxidant system on the ocular surface, reduced the content of MDA, and finally decreased the levels of IL‐1β, IL‐6, and TNF‐α in corneal epithelium.^[^ ^119^ ^]^

Crocin is a water‐soluble carotenoid derived from saffron. It has been ascertained that crocin can augment the activities of SOD, GPX, CAT, and GSH while diminishing oxidative damage products like MDA.^[^ ^120^ ^]^ Qin et al. devised liposome‐encapsulated dual‐drug eye drops incorporating crocin and CsA. Its ROS scavenging capacity curbed the expressions of downstream NF‐κB, p‐P65, and P65, and curtailed the secretion of pro‐inflammatory factors. Meanwhile, crocin mitigated the irritation of CsA on the corneas of DED mice, as CsA was shown to inhibit T cells but potentially elevate the ROS level in normal cells.^[^ ^121^ ^]^

## 5 Discussion

With the changes in modern social environment and lifestyle, the incidence of DED has been rising annually and it has become a globally widespread eye disease. The oxidative damage on the ocular surface should not be underestimated.

**Figure 3:**
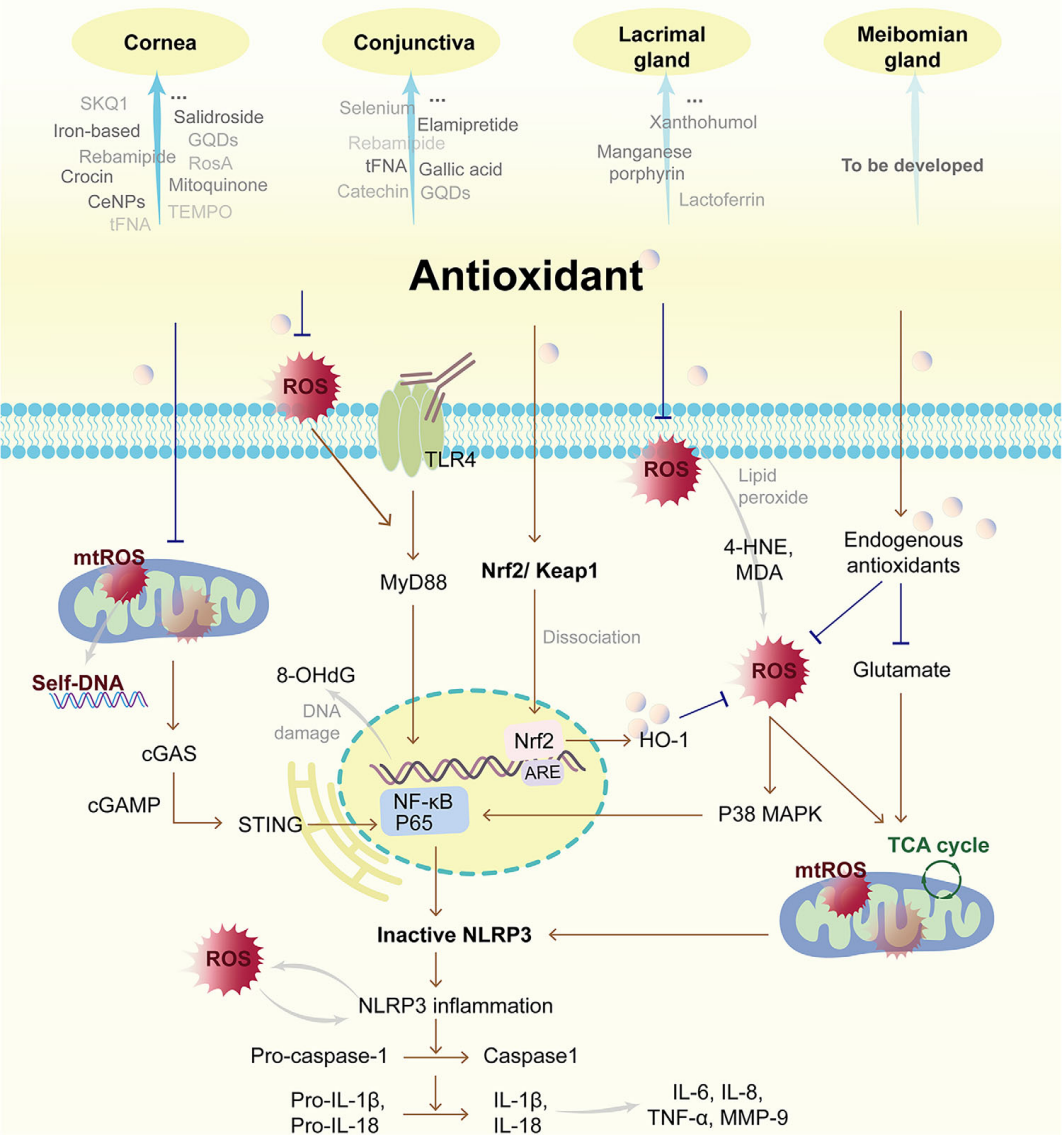
Target sites and mechanisms of antioxidant DED treatment.

In this review, we have elucidated how OS induces and aggravates DED, and systematically summarized the changes in the expressions of enzymatic and nonenzymatic antioxidants as well as the production of various oxidative damage biomarkers in samples of the cornea, TF, LGs, conjunctiva, and MG (Table 1). In the pathology of DED, corneal cells exhibit abnormal histological arrangements, with reduced cellular density and fewer microvilli. The acini, ducts, and nerves of the LGs are impaired, and lipofuscin accumulation can be observed. In the conjunctiva, GCs generation is significantly suppressed, resulting in diminished mucin. The MG also experiences glandular atrophy and fibrosis. Eventually, the integrity of TF is disrupted, leading to decreased tear secretion and shortened TF break‐up time. In all samples, there are reduced numbers of antioxidants and a large increase in oxidized products of lipids, DNA, and proteins. Among these, corneal experiments are more comprehensive, yet there are relatively few studies exploring the relationship between OS changes in the MG and DED. But this does not mean MG is the least affected by OS during DED. We believe the difficulty in sampling may be part of the reason. The MGs have a small diameter and are deeply located, while the eyelid is thin and sensitive. Applying too much pressure on the eyelid may damage or contaminate MG tissues. The available sample size is reduced, which is not conducive to experimental analysis. Moreover, the components of meibum are very complex. Currently, immortalized human MG epithelial cells can only produce the differentiation marker Meibocyte, yet cannot accurately reflect the mature or super‐mature cells required for the holocrine acini in vivo.^[^ ^122^ ^]^ Therefore, in the context of DED studies, most of the antioxidant interventions have primarily focused on repairing the cornea, LGs, and conjunctiva, rather than enhancing the function of the MG and lipid secretion (**Table** **2** and **Figure** **3**). Also, although all antioxidants increase tear secretion, there is relatively little research on mucin secretion.

**Table 2:** Target sites and mechanisms of antioxidant DED treatment.

Antioxidant components	Target sites	Potential mechanism	References
SKQ1	Cornea	↓ ROS, NLRP3, Caspase‐1, IL‐1β	[31, 88]
Rebamipide	Cornea; Conjunctiva	↑ MUC1, MUC4, MUC16, MUC5AC	[90, 91]
Mitoquinone	Cornea	↓ ROS (H2O2), NLRP3, IL‐1β	[93]
Elamipretide	Cornea; Conjunctiva	↑ GPX; ↓ ROS, Glutamate, IL‐1β, IL‐6, TNF‐α	[17]
SOD	Cornea; Conjunctiva	↑ SOD; ↓ ROS, MDA	[23]
TEMPO	Cornea	↓ ROS (H2O2), P38 MAPK, NF‐κB p65, IL‐1β, MMP‐9	[7, 95]
Lactoferrin	Cornea; LGs; Conjunctiva;	↑ IL‐10; ↓ ROS (H2O2, 8‐OHdG, IL‐1β, TNF‐α	[45, 97, 98, 99, 100]
CeNPs	Cornea; Conjunctiva	↑ SOD, CAT, GPX; ↓ ROS (H2O2, O2•−, OH•, TLR4, MyD88, NF‐κB, IL‐1β, TNF‐α	[3, 73, 102, 103, 104, 105, 106]
Iron‐based nanozymes	Cornea; Conjunctiva	↑ SOD, CAT, GPX, HO‐1, IL‐10; ↓ ROS (H2O2, O2•−, OH•, NF‐κB p65, NLRP3, IL‐1β, IL‐6, IL‐18, TNF‐α	[21, 25, 108]
Manganese‐porphyrin	Cornea; LGs; Conjunctiva;	↓ ROS, 8‐OHdG	[5]
Selenium‐based nanozymes	Cornea; Conjunctiva	↑ SOD, CAT, GPX, HO‐1, Nrf2; ↓ ROS (H2O2, OH•), 3‐NT, P38 MAPK, IL‐1β, IL‐8	[27, 110]
GQDs	Cornea; Conjunctiva	↑ Nrf2; ↓ ROS, Keap1, IL‐1β, IL‐6, IL‐8, TNF‐α, IFN‐γ	[111]
tFNA	Cornea; Conjunctiva	↑ SOD; ↓ ROS, 3‐NT, NF‐κB p65, IL‐6, MMP‐9	[18]
Xanthohumol	Cornea; LGs	↑ Nrf2; ↓ 8‐OHdG	[29]
Catechin	Cornea; Conjunctiva	↑ MUC5AC; ↓ ROS, NF‐κB, IL‐6, TNF‐α	[10]
Rosmarinic acid	Cornea	↑ MUC1; ↓ ROS, NF‐κB p65, IL‐1β, MMP‐9	[116]
Gallic acid	Cornea; Conjunctiva	↑ HO‐1, NQO1, Nrf2; ↓ ROS, NF‐κB p65, IL‐1β, IL‐6, TNF‐α	[118]
Salidroside	Cornea	↑ SOD, CAT, HO‐1, NQO1, Nrf2; ↓ ROS, MDA, IL‐1β, IL‐6, TNF‐α	[119]
Crocin	Cornea	↓ ROS, NF‐κB p65, IL‐1β, IL‐6, TNF‐α	[121]

Beyond directly impairing the ocular surface, the impact of OS on DED is also manifested in triggering inflammation and forming a vicious cycle therewith, impeding recovery. According to our review, antioxidant strategies for DED can not only scavenge ROS at the source and augment endogenous antioxidant capacity but also exert a certain inhibitory effect on the expression of pro‐inflammatory factors. From the molecular mechanism perspective, the key pathways involved are activating the Nrf2/HO‐1 pathway and suppressing the ROS/NLRP3/IL‐1β pathway (Figure 3). For instance, rebamipide, N‐GQDs, xanthohumol, gallic acid, and salidroside eye drops facilitate the translocation of Nrf2 into the nucleus, while SKQ1, Mitoquinone, cerium oxide, iron‐based nanozymes, and catechin eye drops reduce the nuclear transcription of NF‐κB. Interrupting the signal transduction between the OS and the NLRP3‐mediated inflammatory response is of great significance. Additionally, inflammatory pathways mediated by TLR4 have also been involved. It conveys an important message: the combination of antioxidant and anti‐inflammatory agents might represent the future direction for DED treatment. In this respect, Chinese herbal medicines possessing multi‐target regulatory functions, along with nanocarriers capable of loading multiple drugs, have emerged as the focal points. Currently, the curative effects of flavonoids and phenolic acid monomers on DED are the most definite, which may be related to their o‐dihydroxyphenyl and phenolic hydroxyl structures. Besides, nanozymes that simultaneously possess properties of catalytic enzymatic and nanomaterial have shown great advantages, including CeNPs, iron nanozymes, manganese‐porphyrin and selenium nanozymes. They are more stable than natural antioxidases and can encapsulate other active ingredients. GQD and tFNA also have such potential.

There is a perspective that OS is involved throughout the DED pathology. Consequently, the duration of antioxidant action in vivo becomes particularly critical. The eyeball is very special. It has a dual protection system, both static and dynamic, to maintain the stability of the intraocular environment, but this also hinders drug delivery. First, the rapid renewal of tears and the drainage through the nasolacrimal duct will lead to a decrease in drug concentration and a shortening of the drug retention time.^[^ ^106^ ^]^ Second, the tight junctions of corneal epithelial cells directly affect drug penetration. As a result, the bioavailability of traditional ocular drug administration is less than 5%.^[^ ^17^, ^21^, ^123^, ^124^ ^]^ Nanoparticles can bypass the physiological barriers and reach the sites where OS and inflammation take place. In this review, antioxidant nanoparticles prepared by charge self‐assembly, like SKQ1 and mitoquinone nanoparticles, have witnessed significant optimization in terms of drug targeting and action time. The antioxidants combined with nano‐sustained‐release delivery systems also exhibit stronger control over DED. Furthermore, more dosage forms beyond eye drops can be developed by leveraging nanosystems, such as CLs loaded with lactoferrin, CeNPs, and PBNPs. In 2021, the FDA approved the first drug‐loaded CLs for marketing.^[^ ^125^ ^]^ Additionally, scleral lenses, punctal plugs, and nasal sprays also serve as potential carriers. Among these, nasal sprays containing cationic nanoemulsions are thought to activate acetylcholine receptors on intranasal trigeminal parasympathetic nerve endings, thereby regulating corneal, conjunctival, and LG function to promote tear secretion and optimize the TF.^[^ ^126^, ^127^ ^]^ It avoids direct ocular irritation and offers more safety. Andrew E. Y.’s team confirmed in animal studies that the bioavailability of nasal spray is significantly higher than that of eye drops and intraperitoneal injections.^[^ ^128^ ^]^ We believe that the integration of nanotechnology with antioxidants holds great promise in DED treatment, potentially delivering enhanced benefits to patients.

## 6 Conclusion

As an upstream mechanism, OS exerts a profound influence on the pathogenesis of DED. Antioxidant therapy is conducive to improving the structure and function of the cornea, LGs, conjunctiva, and MG. Therefore, it can maintain the stability of the TF, reverse the THO, and suppress inflammation. Attaching importance to the potential of traditional Chinese medicine and nano‐preparations and developing more efficient, convenient, and safe antioxidant regimens should be an important part of the multi‐target treatment of DED.

## Conflict of Interest

The authors declare no conflict of interest.

## Author Contributions

R.H. wrote the main manuscript text and prepared all the figures. J.S. and C.‐M.X. prepared the investigation and tables. Y.X.L. is in charge of supervision. All authors reviewed the manuscript.
